# Protective role of crocin against sepsis-induced injury in the liver, kidney and lungs via inhibition of p38 MAPK/NF-κB and Bax/Bcl-2 signalling pathways

**DOI:** 10.1080/13880209.2022.2042328

**Published:** 2022-02-27

**Authors:** Jun Gao, Feng Zhao, Shaona Yi, Shuhang Li, Aiqing Zhu, Yingxiu Tang, Aiqun Li

**Affiliations:** a Department of Laboratory Medicine, Yantai Affiliated Hospital of Binzhou Medical University, Yantai, China; b Department of Nephrology, Yantai Affiliated Hospital of Binzhou Medical University, Yantai, China; c Department of Urology, Yantai Affiliated Hospital of Binzhou Medical University, Yantai, China; d Department of Dermatology, Yantai Affiliated Hospital of Binzhou Medical University, Yantai, China; e Department of Emergency, Yantai Affiliated Hospital of Binzhou Medical University, Yantai, China

**Keywords:** Anti-inflammatory, antiapoptotic, antioxidant, organ

## Abstract

**Context:**

Crocin has been reported to have multiple bioactivities. However, the effect of crocin administration on caecal ligation and puncture (CLP)-induced sepsis remains unknown.

**Objective:**

We investigated the effects of crocin on CLP-induced sepsis in mice and the underlying mechanism of action.

**Materials and methods:**

Five experimental groups (*n* = 10) of BALB/c mice were used: control, CLP (normal saline) and CLP + crocin (50, 100 and 250 mg/kg, 30 min prior to CLP). Mice were sacrificed 24 h after CLP. Liver, kidney and lung histopathology, indicator levels, apoptotic status, pro-inflammatory cytokines and relative protein levels were evaluated.

**Results:**

Compared to the CLP group, crocin treatment significantly increased the survival rate (70%, 80%, 90% vs. 30%). Crocin groups exhibited protection against liver, kidney and lung damage with mild-to-moderate morphological changes and lower indicator levels: liver (2.80 ± 0.45, 2.60 ± 0.55, 1.60 ± 0.55 vs. 5.60 ± 0.55), kidney (3.00 ± 0.71, 2.60 ± 0.55, 1.40 ± 0.55 vs. 6.20 ± 0.84) and lungs (8.00 ± 1.59, 6.80 ± 1.64, 2.80 ± 0.84 vs. 14.80 ± 1.79). The proinflammatory cytokines (IL-1β, TNF-α, IL-6 and IL-10 levels in the crocin groups) were distinctly lower and the apoptotic index showed a significant decrease. Crocin administration significantly suppressed p38 MAPK phosphorylation and inhibited NF-κB/IκBα and Bcl-2/Bax activation.

**Discussion and conclusions:**

Pre-treatment with crocin confers protective effects against CLP-induced liver, kidney and lung injury, implying it to be a potential therapeutic agent.

## Introduction

Sepsis, which is classified as a complex syndrome triggered by infection, is widely considered as one of the major causes of death in intensive care units (ICUs) (Rello et al. 2017; Gharamti et al. 2021; Yu et al. 2021). Severe sepsis can affect multiple systems and organs; even after treatment, patients with sepsis face long-term and serious problems. In recent years, numerous medical technologies to advance the diagnosis and treatment of sepsis have been developed; however, clinical methods to avoid and treat sepsis are still limited (Lin GL et al. 2018). Thus, the investigation of mechanism and the discovery of effective medicines and treatment methods for treating septic injury will be helpful (Chang 2019; Huang M et al. 2019).

More recent studies have confirmed that the effects of sepsis comprise systemic inflammatory response and complex immunopathological mechanisms (Englert et al. 2019). Cell inflammation, cytokine production and abnormal apoptosis are also involved in the pathogenesis of sepsis (Gille-Johnson et al. 2013; Teoh et al. 2013). Previous studies have demonstrated that the overactivated inflammatory response in sepsis is the leading cause of organ physiological, pathological and biochemical abnormalities (Cho et al. 2017; Chousterman et al. 2017; Minasyan 2017; Huang M et al. 2019). Therefore, carrying out research on developing potential drug candidates that are capable of repressing inflammatory responses and cell apoptosis-induced septic organ injury is essential for sepsis therapy (Brahmbhatt et al. 2005; Guo et al. 2012; Hu et al. 2021).

Crocin, a carotenoid compound isolated from saffron (*Crocus sativus* L.) and gardenia (*Gardenia jasminoides* Ellis) plants, may be used to treat and prevent many diseases (Alavizadeh and Hosseinzadeh 2014; Lin L et al. 2019; Hadipour et al. 2021). There is accumulating evidence that crocin demonstrates a variety of bioactivities such as antitumor, antioxidant, anti-inflammatory, anti-hyperlipidaemic, anti-atherosclerotic, free-radical scavenging and neuroprotective activities (Naghizadeh et al. 2008; Mashmoul et al. 2016; Razmaraii et al. 2016; Kocaman et al. 2019; Omidkhoda et al. 2020; Khanmohammadi et al. 2021). Through its anti-inflammatory and antioxidant properties, treatment with crocin alleviates organ (e.g., the lungs, heart, brain and kidney) injury by regulating the NF-κB and PI3K/Akt pathways (Lari et al. 2015). Other studies have shown that crocin also has antioxidant and anti-inflammatory effects against ischemia–reperfusion damage in isolated rat hearts (El-Kharrag et al. 2017; Chhimwal et al. 2020). Crocin administration 30 min prior to LPS administration could prevent endotoxin-mediated inflammation, prevent LPS-induced sepsis and improve the clinical status of animals that are challenged by sepsis (Kim et al. 2014; Baradaran Rahim et al. 2019). Nevertheless, there have been few studies on the effect of crocin on sepsis-induced lung, liver and kidney injury.

As mentioned above, crocin exerts preventive effects on LPS-induced sepsis models because of its antioxidant and anti-inflammatory properties. However, whether crocin has a protective effect against sepsis-induced organ injury remains unclear. Therefore, in the present study, we established an internationally recognized caecal ligation and puncture (CLP) sepsis model to determine the effects and mechanisms of crocin on CLP-induced septic organ injury.

## Materials and methods

### Animals, sepsis model and procedure of experiment

After approval of the Yantai Affiliated Hospital of Binzhou Medical University (approval number: IRB2021-230), all animal experiments were performed according to the National Institutes of Health Guide for the Care and Use of Laboratory Animals. Sixty BALB/C mice (20 ± 2 g) were obtained from Jinan Pengyue Experimental Animal Breeding Co., Ltd. (license number SCXK [Lu] 20170026; Jinan, China). Crocin was obtained from Sigma-Aldrich (St. Louis, MO).

The animals were acclimatized for 1 week prior to experimentation and housed in polycarbonate cages in a standard room (21 ± 3 °C, 45–65% relative humidity and a 12 h light/dark cycle). The animals were fed standard laboratory animal feed and water *ad libitum*.

Mice were randomly divided into five groups (*n* = 10): control group (animals underwent identical laparotomy; the caecum was exposed but not ligated or punctured), CLP-vehicle group (CLP-induced controls treated with normal saline) and three CLP-induced groups treated with crocin at three different doses (50, 100 and 250 mg/kg) 30 min prior to CLP. Crocin doses in our study were selected according to previous studies (Boussabbeh et al. 2016; Vafaei et al. 2020): CLP + CRO 50 (CLP-induced mice administered 50 mg/kg crocin), CLP + CRO 100 (CLP-induced mice administered 100 mg/kg crocin) and CLP + CRO 250 (CLP-induced mice administered 250 mg/kg crocin).

To set up the CLP model, the mice were anaesthetized with 2% isoflurane inhalation. After the abdomen was shaved, a 2 cm incision was made to expose the abdominal organs. The caecum was isolated and ligated 0.5 cm from the tip with a 3-0 silk ligature. The caecum punctures were performed using a 22-gauge needle, extruding a small amount of faecal content. The caecum was then placed back into the peritoneal cavity, and the abdominal incision was sewn up. Sham-operated (control) mice received laparotomies, and the caecum was exposed but not ligated or perforated. Normal saline (1 mL) was applied to the mice immediately after the operation. The CLP method and period were chosen according to previous studies (Aziz et al. 2018). After CLP treatment, survival was assessed at 0, 3, 6, 12 and 24 h after surgery, and the survival rate was recorded.

### Sample collection and homogenate preparation

All animals were sacrificed under anaesthesia, 24 h after CLP. Blood samples, lung tissues, kidney tissues and liver tissues were collected for further analysis. The tissues were cut into several pieces for histological structural analysis, apoptotic evaluation and further biochemical evaluation. Blood samples of the mice were collected immediately and transferred to the laboratory, centrifuged at 3000 *×* *g* for 15 min, and then stored at −20 °C for the detection of inflammatory cytokines and other relevant biochemical parameter levels.

### Histopathological examination

Briefly, formalin-fixed and paraffin-embedded lung, kidney and liver tissue blocks were sectioned at 5 μm thickness and placed on glass slides. Tissue sections were stained with haematoxylin and eosin (H&E) for observation. Pathological changes in tissue sections were evaluated by microscopy using ImageJ and a light microscope (Sigma-Aldrich, St. Louis, MO). Histological changes were scored on a scale of 0 (normal findings), 1 (mild injury), 2 (moderate injury), 3 (significant injury) and 4 (severe injury), and four variables were summed to represent the organ injury, as detailed in previous studies (Aziz et al. 2018; Chen et al. 2020; Malkoç et al. 2020).

### Assessment of cytokine measurement

Inflammatory cytokines, including IL-1β, TNF-α, IL-6 and IL-10, were detected using an enzyme-linked immunosorbent assay (ELISA) kit (eBioscience, San Diego, CA) according to the manufacturer’s instructions.

### Determination of AST, ALT, BUN and creatinine levels and MPO activity

Commercial kits (BioVision, Mountain View, CA) were used to quantify the levels of serum alanine aminotransferase (ALT), aspartate aminotransferase (AST), serum blood urea nitrogen (BUN) and creatinine. Myeloperoxidase (MPO) activity was measured via lung function tests using the MPO colorimetric activity assay kit (BioVision, Mountain View, CA) following the manufacturer’s instructions.

### Measurement of lung, kidney and liver cell apoptosis

Terminal deoxynucleotidyl transferase deoxyuridine triphosphate nick-end labelling (TUNEL) staining assay was used to assess cell apoptosis (Li et al. 2019) using a TUNEL kit (Millipore; Merck KGaA, Darmstadt, Germany). The sections were observed and photographed using an optical microscope (Olympus Corp., Tokyo, Japan), five random fields were selected in each section, and the apoptotic indexes were calculated (Hung et al. 2017).

### Western blotting

Lung, kidney and liver tissues of each group were homogenized on ice, centrifuged and lysed in RIPA lysis buffer. Protein concentrations in all samples were determined using a BCA protein kit. Proteins in the samples were separated using 10% SDS-PAGE and transferred to PVDF membranes. Membranes were blocked with Tris-buffered saline containing 5% non-fat milk and 0.1% Tween-20 for 2 h at room temperature. The membranes were then incubated with primary antibodies (p-p38, p38, p-NF-κB-p65, NF-κB, p-IκBα, IκBα, Bax and Bcl-2; 1:1000 dilution; Cell Signaling Technology, Boston, MA) in blocking buffer overnight at 4 °C. The blots were incubated with a horseradish peroxidase-coupled secondary antibody (1:2000, Cell Signaling Technology, Boston, MA) at 37 °C for 1.5 h and visualized with an electrochemiluminescence system. The protein bands were analysed using Image J software (National Institutes of Health, Bethesda, MD).

### Statistical method

All data were analysed using IBM SPSS Statistics (version 21.0; Chicago, IL) and expressed as mean ± standard deviation (mean ± SD). For survival rate studies, Kaplan–Meier’s analyses followed by log-rank tests were performed. The significant difference of data in multiple groups was analysed by one-way analysis of variance (ANOVA) followed by Tukey’s *post hoc* test, and *p* < 0.05 was considered to be statistically significant.

## Results

### Crocin increased survival rate in septic mice

**Figure 1.**
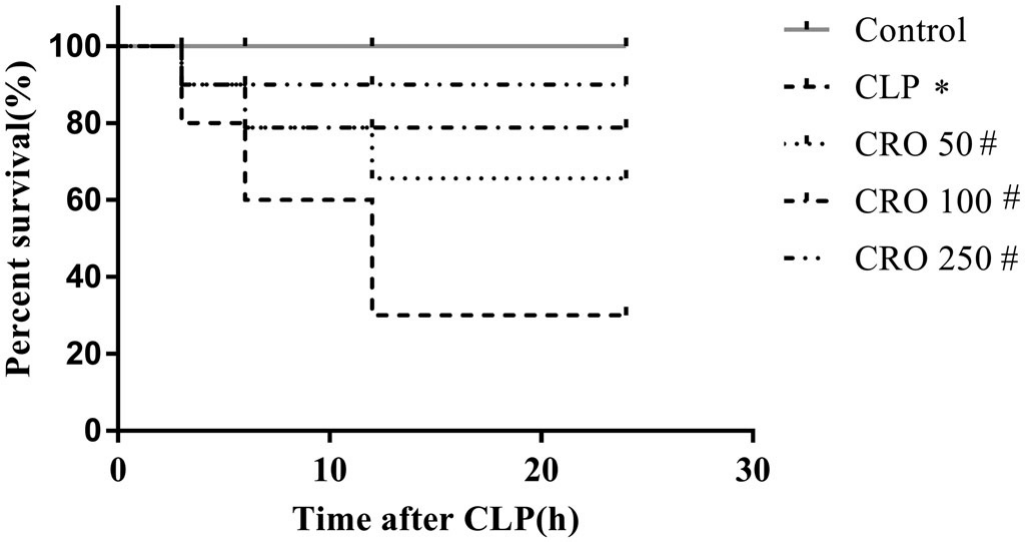
Effect of crocin on survival rate in mice. The survival rates of mice were evaluated within 24 h. *p < 0.05 vs. control group, ^#^*p* < 0.05 vs. CLP group.

To investigate the protective effects of crocin on the survival rate of septic mice, we measured the survival rate at 0, 3, 6, 12 and 24 h after CLP. As shown in Figure 1, compared to the control group, the survival rate of mice in the CLP group was markedly lower than that in the control group (30% vs. 100%, *p* < 0.05). There was no significant difference between the control and CRO groups, and the survival rates in the CRO groups were distinctly higher than those in the CLP group (70%, 80%, 90% vs. 30%, *p* < 0.05). These results suggest that crocin treatment could improve the survival rate. Moreover, the CRO 250 group exhibited a significantly increased survival rate (Figure 1).

### Crocin ameliorated lung, liver and kidney injury

To determine the effects of crocin on sepsis-induced lung, liver and kidney injury, we performed H&E staining to observe the morphology of the tissues. The lungs, liver and kidneys in the control group had normal morphological characteristics.

**Figure 2.**
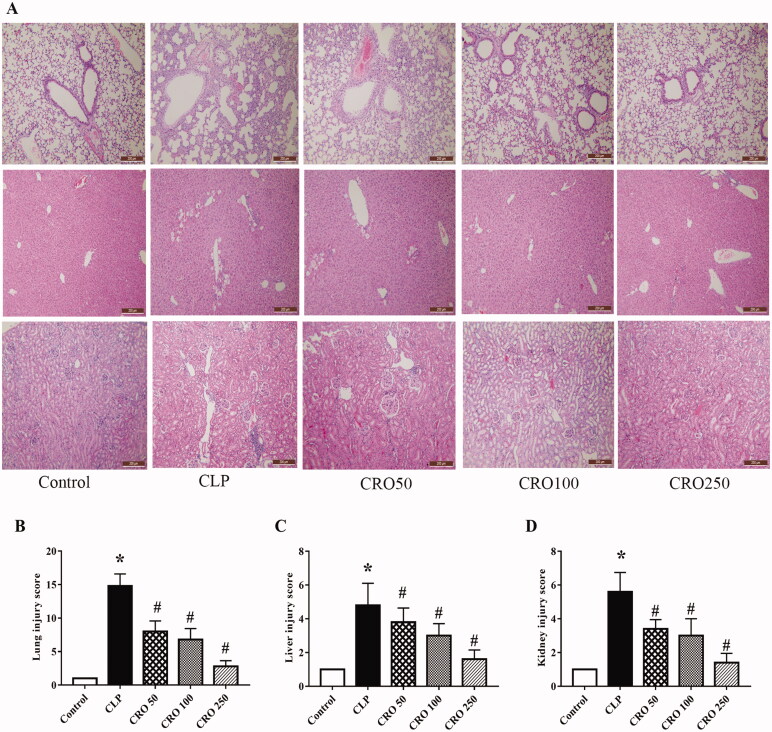
Effect of crocin on histopathological changes in lung, liver and kidney tissues in mice. (A) Histopathological changes in mouse lung, liver and kidney tissue, observed via H&E staining (magnification ×100). (B) Lung injury scores of mice. (C) Liver injury scores of mice. (D) Kidney injury scores of mice. *p< 0.05 vs. control group, ^#^*p*< 0.05 vs. CLP group.

Compared with the control group, the lung alveolar wall thickness was larger and the number of pulmonary alveoli was lower in the CLP group. In addition, histological images of lung tissue in the CLP group showed alveolar congestion, exudate, inflammatory cell infiltration and severe alveolar-capillary structure damage. However, compared with CLP group, administration of crocin repressed alveolar wall swelling and attenuated the decline in the number of pulmonary alveoli, alveolar congestion, amount of exudate, and inflammatory cell infiltration in the CRO groups (Figure 2(A)). Compared with control group, lung histological change scores were higher in the CLP group (*p* < 0.05). In contrast, we found no significant difference between the control and CRO groups. Compared with CLP group, lung tissue scores in the CRO groups were significantly lower (8.00 ± 1.59, 6.80 ± 1.64, 2.80 ± 0.84 vs. 14.80 ± 1.79, *p* < 0.05, Figure 2(B)). Moreover, CRO 250 group exhibited a significant decrease in the lung tissue scores of CLP mice after treatment.

Liver histopathology showed morphological alterations in hepatic features, necrosis and tissue degeneration compared with the control group. As shown in Figure 2(A), liver tissue exhibited aggregation of degeneration, inflammation and focal necrosis of hepatocytes. However, compared with the CLP group, the crocin groups showed an improvement in hepatic alterations. Compared with the control group, histological change scores in the liver were significantly higher in the CLP group (*p* < 0.05). No difference was observed between the crocin and control groups. Compared with CLP group, liver tissue scores in the crocin groups were significantly lower (2.80 ± 0.45, 2.60 ± 0.55, 1.60 ± 0.55 vs. 5.60 ± 0.55, *p* < 0.05, Figure 2(C)). Moreover, in the CRO 250 group, treatment significantly decreased the liver tissue scores of CLP mice.

In kidney histopathology, compared with the control group, the CLP group showed morphological alterations in renal features, including glomerular swelling and injury and inflammatory cells in glomeruli. However, compared with the CLP group, the crocin groups showed an improvement in renal features. Histological change scores in the kidney were significantly higher in the CLP group than in the control group (*p* < 0.05). In contrast, we found no significant difference between the control and CRO groups. Kidney tissue scores in the CRO groups were significantly lower (3.00 ± 0.71, 2.60 ± 0.55, 1.40 ± 0.55 vs. 6.20 ± 0.84, *p* < 0.05, Figure 2(D)). Moreover, in the CRO 250 group, treatment significantly decreased the kidney tissue scores of CLP mice.

Treatment with crocin improved the histological change scores in lung, liver and kidney tissues and led to an improvement in lung, liver and kidney damage with mild-to-moderate morphological changes.

### Crocin improved apoptotic indexes

We evaluated apoptotic indexes to determine the degree of lung injury (MPO activity), liver injury (ALT and AST levels) and kidney injury (BUN and creatinine levels) induced by CLP.

**Figure 3.**
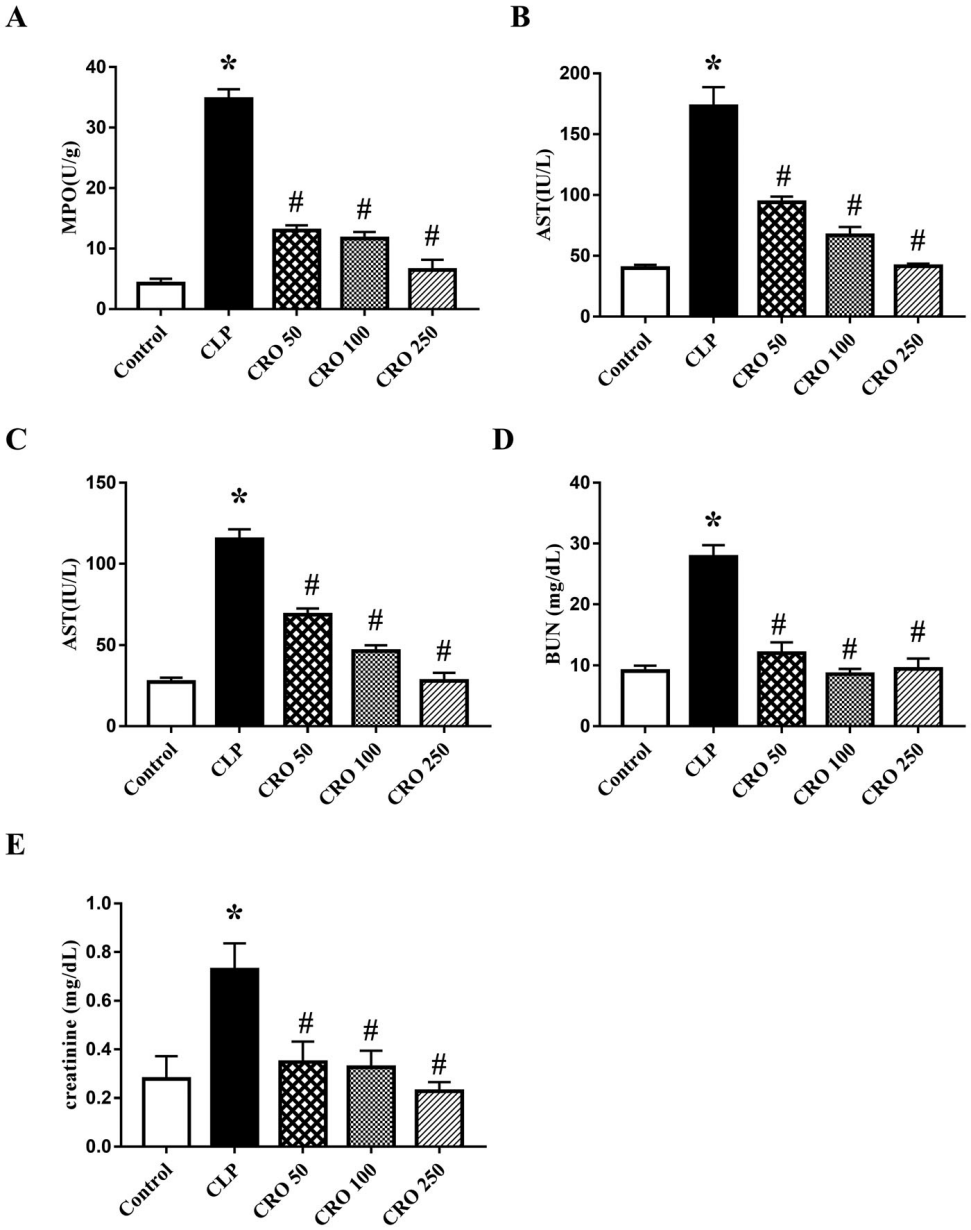
Effect of crocin on myeloperoxidase (MPO) activity and aspartate aminotransferase (AST), serum alanine aminotransferase (ALT), serum blood urea nitrogen (BUN) and creatinine levels. (A) MPO activity was measured using the MPO colorimetric activity assay kit. (B) AST levels were measured using the AST colorimetric activity assay kit. (C) ALT levels were measured using the ALT colorimetric activity assay kit. (D) BUN levels were measured using the BUN colorimetric activity assay kit. (E) Creatinine levels were measured using the creatinine colorimetric activity assay kit. *p< 0.05 vs. control group, ^#^*p*< 0.05 vs. CLP group.

As expected, compared with the control, the MPO activity and levels of ALT, AST, BUN and creatinine in the CLP group were markedly higher (*p* < 0.05). In contrast, we found no significant difference between the control and CRO groups. The indexes in the CRO groups were significantly lower than those in the CLP group (*p* < 0.05, Figure 3). The results showed that crocin attenuated the indexes, indicating alleviation of the degree of injury in lung, liver and kidney tissues and mitigation of CLP-induced tissue injury.

### Crocin decreased apoptosis in lung, liver and kidney tissues

**Figure 4.**
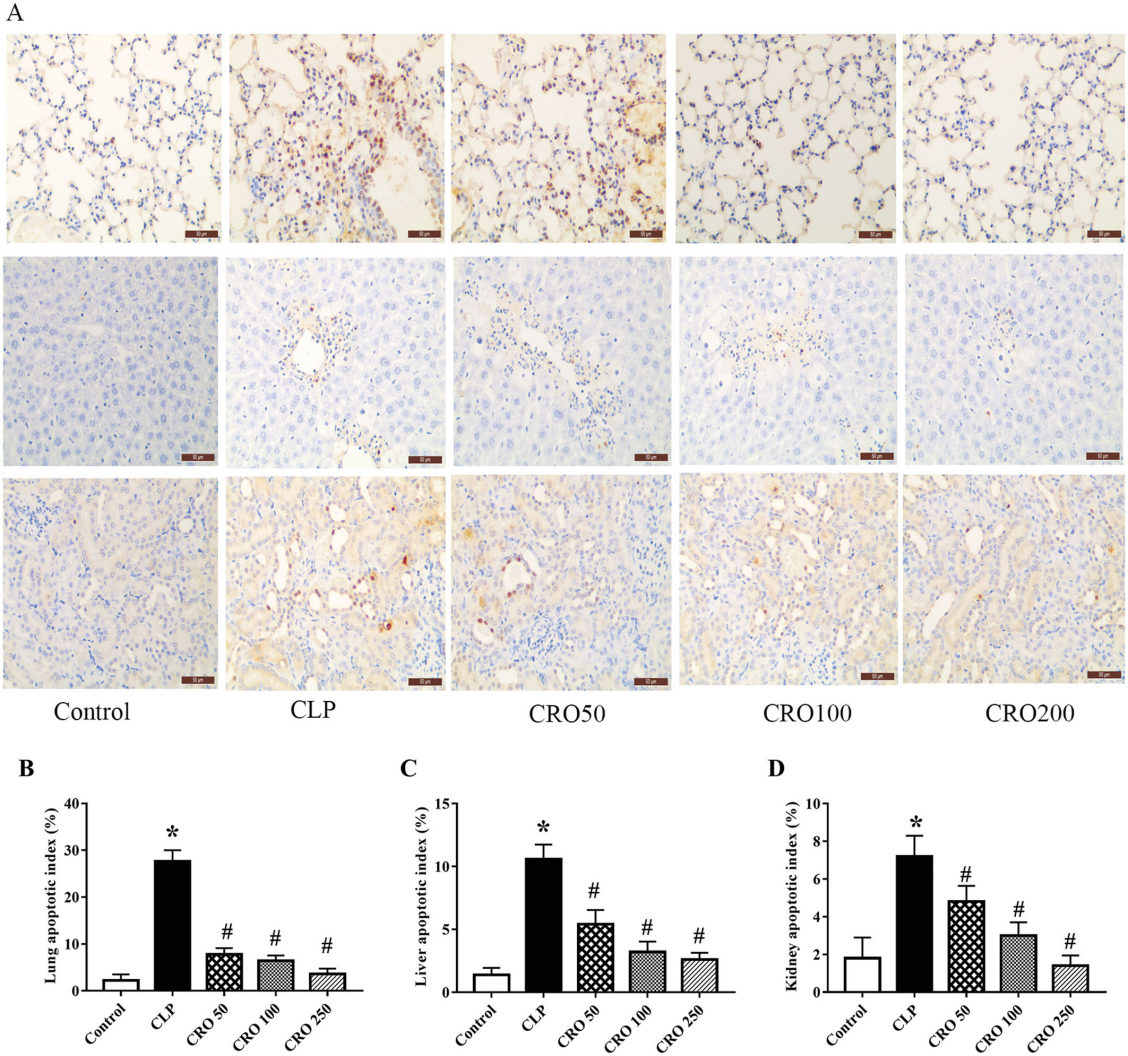
Effect of crocin on cell apoptosis in lung, liver and kidney tissues of mice. Apoptosis was detected via TUNEL staining (×400) in each case. (A) Lung, liver and kidney cell apoptosis. (B) Quantitative data of lung cell apoptosis index. (C) Quantitative data of liver cell apoptosis index. (D) Quantitative data of kidney cell apoptosis index. *p< 0.05 vs. control group, ^#^*p*< 0.05 vs. CLP group.

TUNEL-stained photomicrographs, as shown in Figure 4, were used to determine the effects of crocin on apoptosis in lung, liver and kidney tissues. As shown in Figure 4, compared with the control group, the apoptotic index was markedly increased in CLP group (*p* < 0.05; Figure 4). There was no significant difference between the control and CRO groups. Apoptotic indexes were significantly lower in the CRO group than in the CLP groups (*p* < 0.05; Figure 4). The results showed that crocin treatment in the CRO groups attenuated the apoptotic indexes in lung, liver and kidney tissues.

### Crocin inhibited inflammatory response

**Figure 5.**
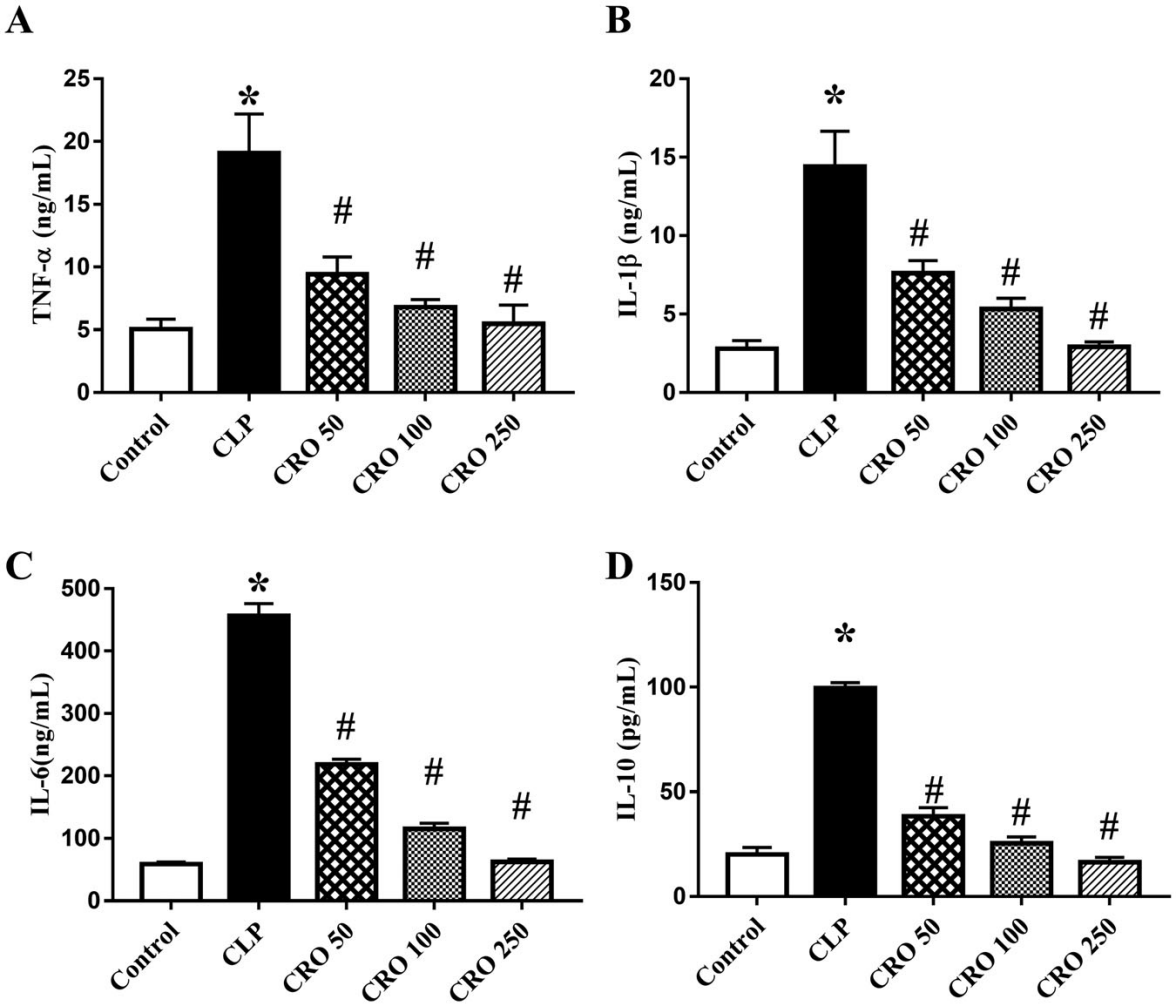
Effect of crocin on serum levels of inflammatory factors, as quantified via enzyme-linked immunosorbent assay (ELISA), in mice. (A) IL-1β levels. (B) TNF-α levels. (C) IL-6 levels. (D) IL-10 levels. *p< 0.05 vs. control group, ^#^*p*< 0.05 vs. CLP group.

IL-1β, TNF-α, IL-10 and IL-6 are pro-inflammatory cytokines that play core roles in the pathogenesis of sepsis. To determine the effects of crocin on inflammation, we measured the levels of IL-1β, TNF-α, IL-6 and IL-10. As shown in Figure 5, the levels of IL-1β, TNF-α, IL-6 and IL-10 in the CLP group were markedly higher than those in the control group (*p* < 0.05), while there was no difference between the control and CRO groups. Levels of pro-inflammatory cytokines in the CRO groups were distinctly lower than those in the CLP group (*p* < 0.05). These results suggest that pre-treatment with crocin decreased the production of IL-1β, TNF-α, IL-6 and IL-10 in a concentration-dependent manner.

### Crocin decreased the phosphorylation of p38 MAPK and suppressed activation of NF-κB

To further investigate the mechanism of action of crocin on CLP-induced damage, we examined the expression levels of Bcl-2, Bax, NF-κB-p65, IκBα and p38 in lung, liver and kidney tissues.

**Figure 6.**
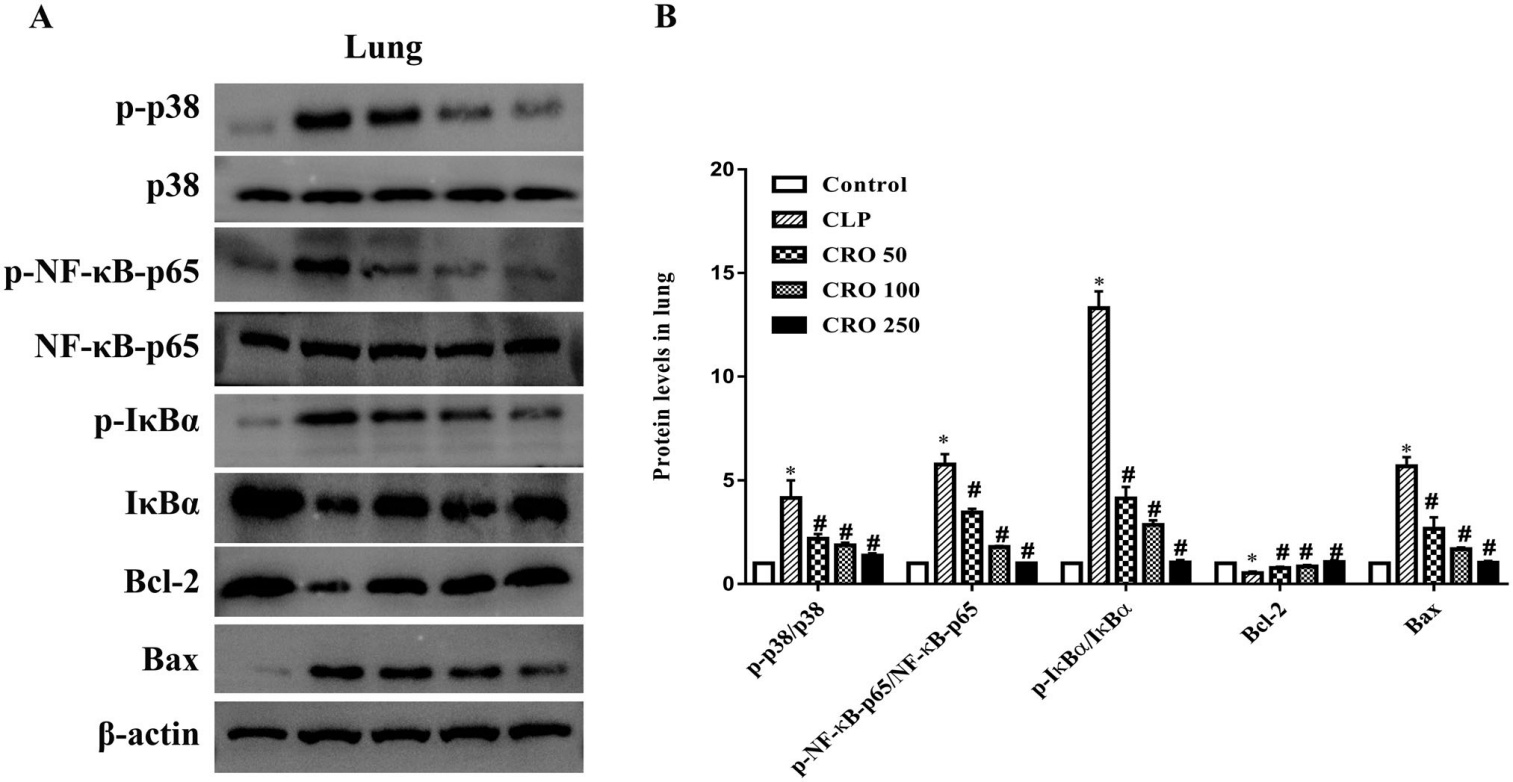
Effect of crocin on the p38MAPK/NF-κB and Bcl-2/Bax pathways in lungs of mice. (A) Expression levels of p-p38, p38, p-NF-κB-p65, NF-κB-p65, p-IκBα, IκBα, Bcl-2 and Bax, as quantified via western blot analysis. (B) Quantitative data of the levels of p-p38/p38, p-NF-κB-p65/NF-κB-p65, p-IκBα/IκBα, Bcl-2 and Bax. *p< 0.05 vs. control group, ^#^*p*< 0.05 vs. CLP group.

**Figure 7.**
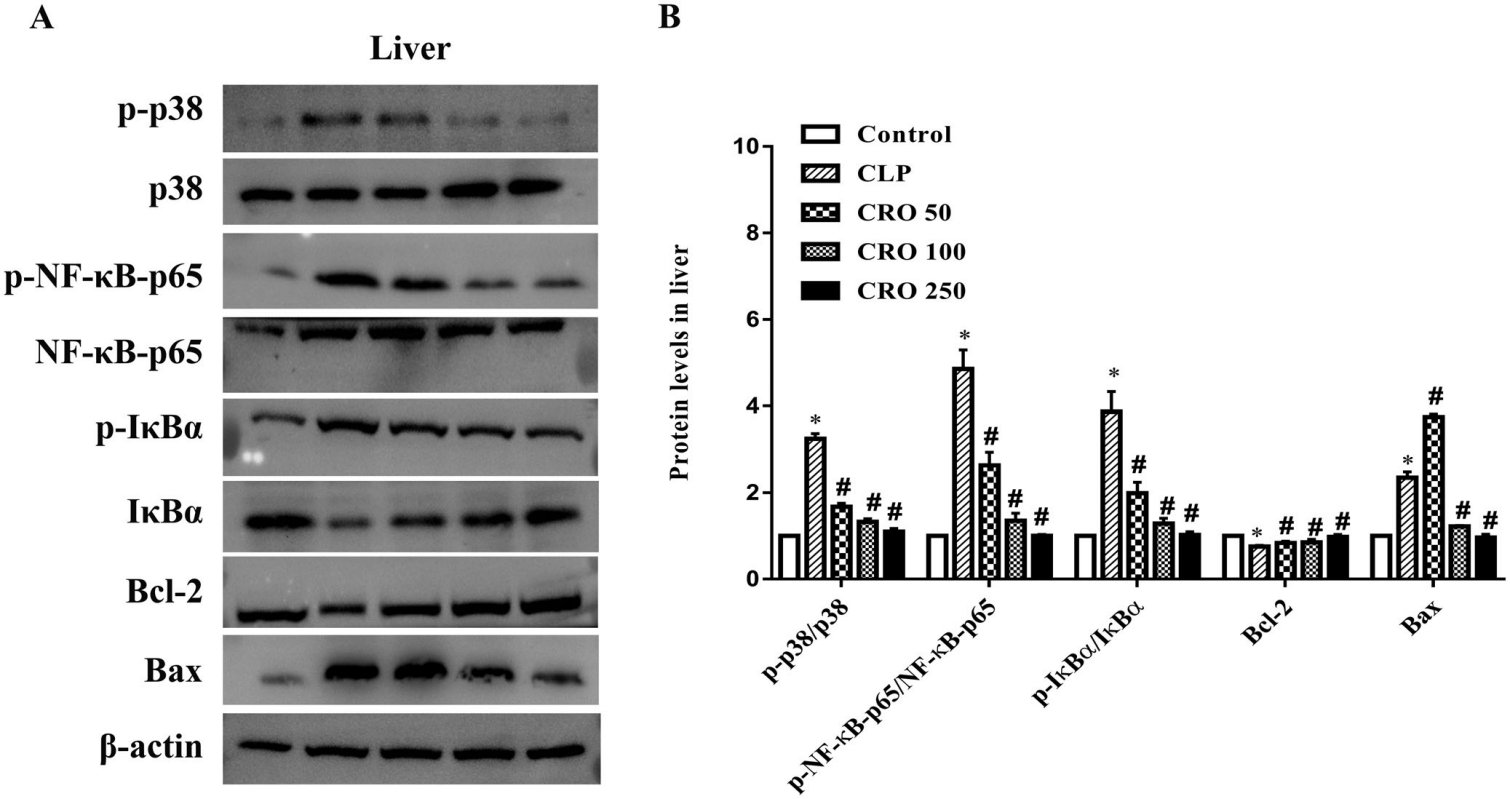
Effect of crocin on the p38MAPK/NF-κB and Bcl-2/Bax pathways in livers of mice. (A) Expression levels of p-p38, p38, p-NF-κB-p65, NF-κB-p65, p-IκBα, IκBα, Bcl-2 and Bax, as quantified via western blot analysis. (B) Quantitative data of the levels of p-p38/p38, p-NF-κB-p65/NF-κB-p65, p-IκBα/IκBα, Bcl-2 and Bax. *p< 0.05 vs. control group, ^#^*p*< 0.05 vs. CLP group.

**Figure 8.**
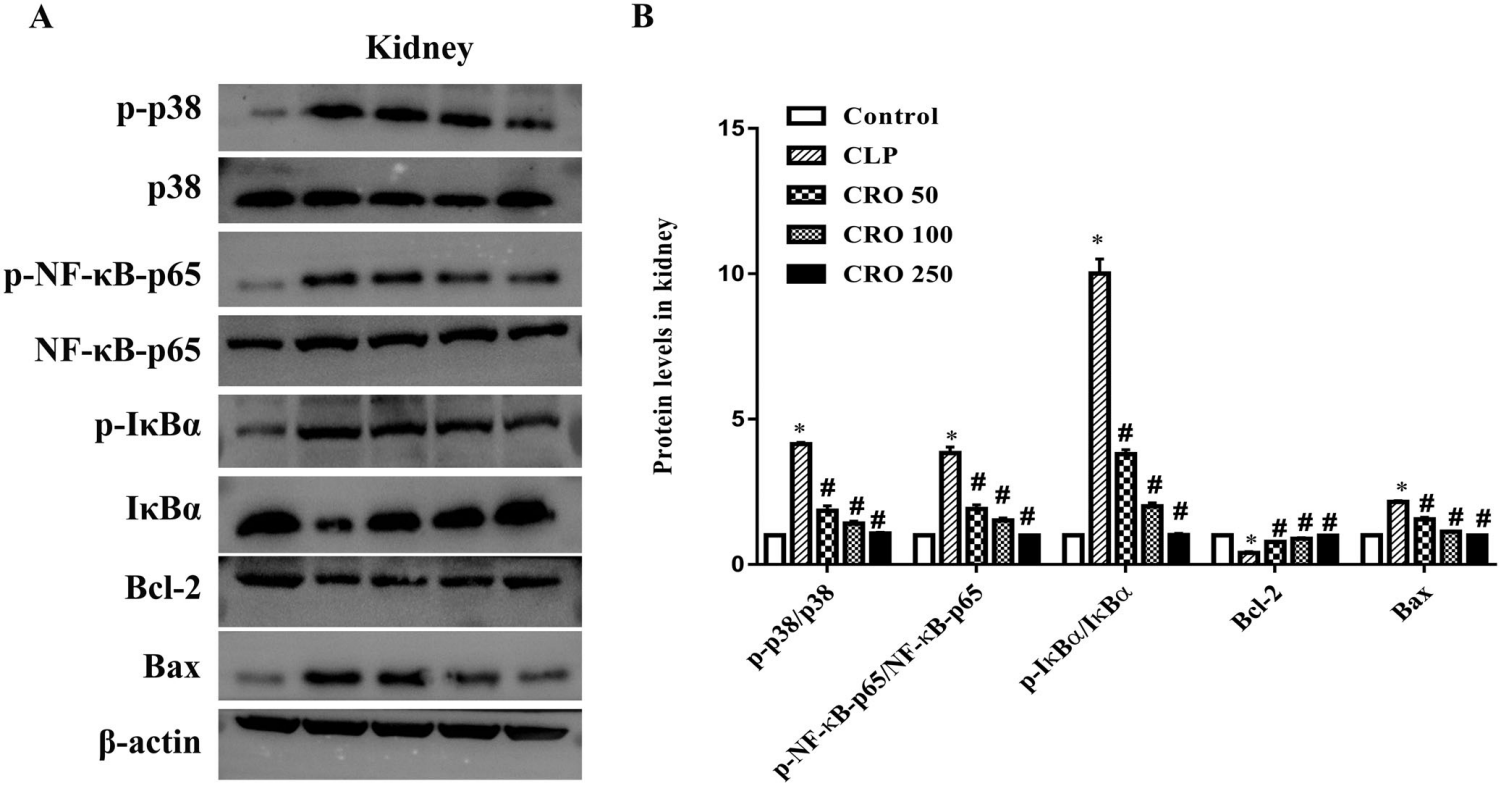
Effect of crocin on the p38MAPK/NF-κB and Bcl-2/Bax pathways in kidneys of mice. (A) Expression levels of p-p38, p38, p-NF-κB-p65, NF-κB-p65, p-IκBα, IκBα, Bcl-2 and Bax, as quantified via western blot analysis. (B) Quantitative data of the levels of p-p38/p38, p-NF-κB-p65/NF-κB-p65, p-IκBα/IκBα, Bcl-2 and Bax. *p< 0.05 vs. control group, ^#^*p*< 0.05 vs. CLP group.

As shown in Figures 6–8, compared with the control group, the ratios of expression levels, specifically, p-p38/p38, p-NF-κB-p65/NF-κB-p65 and p-IκBα/IκBα, were markedly higher in the CLP group (*p* < 0.05). No difference was observed between the crocin and control groups. Compared with the CLP group, treatment with crocin significantly decreased CLP-induced p-p38/p38, p-NF-κB-p65/NF-κB-p65 and p-IκBα/IκBα (*p* < 0.05).

Compared with the control group, the CLP group exhibited upregulation of Bax expression and downregulation of Bcl-2 expression in lung, liver and kidney tissues. No difference was observed between the crocin and control groups. When compared with the CLP group, the expression level of Bax was significantly decreased and that of Bcl-2 was markedly increased by administration of crocin (50, 100 and 250 mg/kg).

## Discussion

Although some studies have reported that crocin has several biological activities such as antioxidative, antitumor and anti-inflammatory activities, the effects of crocin on CLP-induced sepsis remain unclear (Boussabbeh et al. 2016; Lin L et al. 2019). Severe sepsis is known to induce multiple organ failure alterations, such as organ histopathology parameters and biochemical and haematological alterations (Minasyan 2017; Aziz et al. 2018). Notably, complex factors, such as inflammation, activation of the complement system, and increased oxidative stress may induce multiple organ failure (Heung and Koyner 2015).

In our study, sepsis was induced in mice by CLP (Aziz et al. 2018). To investigate the protective effect of crocin on sepsis-induced organ injury, we studied the histopathological features and injury indexes of the lung, liver and kidney. These findings suggest that after the CLP procedure, the CLP group showed remarkable lung architectural changes and an increase in MPO activity, while crocin groups showed mild-to-moderate necrotic changes. The crocin groups showed a protective effect against lung tissue injuries, while MPO activity was significantly decreased. In terms of its anti-inflammatory and antioxidant properties, crocin has been reported to decrease liver damage in mice (Yaribeygi et al. 2018; Kalantar et al. 2019). Similarly, our results revealed liver histological alteration of renal features accompanied by a significant increase in serum ALT and AST concentrations in crocin groups. Moreover, our results showed that crocin protected against kidney damage, while reducing creatinine and BUN levels, which is consistent with the results of previous studies (Zaghloul et al. 2019).

Excessive accumulation of inflammatory factors is a common mechanism of sepsis. According to several studies, macrophages are widely distributed in the body and are able to release a large number of inflammatory factors, such as TNF-α, IL-1β and pro-inflammatory mediators. In addition, macrophages play a key role in the regulation of the inflammatory response and regulation of the pathogenesis of sepsis. During the early stage of CLP-induced sepsis, the body activates inflammatory responses and secretes early pro-inflammatory mediators, such as TNF-α and IL-1β, to protect against infection. Although the inflammatory response is a good way to combat infection, overactivated inflammation and systemic inflammatory responses lead to multiple organ failure or death. In terms of possible inflammatory mechanism, recent studies emphasized that the activation of the MAPK and NF-κB/IκBα signalling pathways plays an important role in regulating the secretion of pro-inflammatory cytokines and mediators (Wang et al. 2020). It has been documented that MAPKs are upstream activators of NF-κB and act as an important signal in regulating activation of NF-κB, which is associated with inflammation and apoptosis in sepsis. It has been proposed that p38 MAPK plays an important role in the activation and migration of NF-κB to the nucleus, thus activating the activity of NF-κB and promoting the expression of pro-inflammatory mediators, such as IL-1β and TNF-α (Huang B et al. 2018; Cao et al. 2020). Hence, inhibition of the activation of MAPK and NF-κB leads to attenuation of the production of pro-inflammatory cytokines and impedes cell apoptosis. In the present study, we examined the phosphorylation of the p38 MAPK protein. Our results demonstrated that crocin decreased the phosphorylation of p38 MAPK and suppressed the activation of NF-κB. The level of NF-κB was consistent with the upregulation of IL-1β, IL-6 and TNF-α expression. These results suggest that treatment with crocin largely enhances survival by activating anti-apoptotic effectors and by inhibiting p38 MAPK phosphorylation and NF-κB activation.

In the context of organ injury and inflammation, cell apoptosis induced by CLP is also involved in sepsis. In the present study, apoptosis in lung, liver and kidney tissues was detected via TUNEL assay, and crocin treatment resulted in a remarkable reduction in apoptotic cells. It has been documented that apoptosis is caused by a series of physiological and pathological signals, while Bcl-2 and Bax are key regulators of the cell pathway (Fu et al. 2014; Klingensmith et al. 2019). To elucidate the mechanism of cell apoptosis, we analysed Bax and Bcl-2 protein expression via western blotting. We observed that crocin prevented organ apoptosis by increasing the expression of anti-apoptotic Bcl-2 and decreasing the expression of pro-apoptotic Bax. Furthermore, the results demonstrated that crocin exerts anti-apoptotic activity through the Bcl-2/Bax signalling pathway.

In summary, crocin exerts anti-inflammatory and anti-apoptotic activities by regulating the p-38 MAPK/NF-κB and Bcl-2/Bax signalling pathways and by reducing levels of pro-inflammatory mediators.

## Conclusions

The current study provides a comprehensive elucidation of the organ-protective effects of crocin. Additionally, the anti-inflammatory and anti-apoptotic effects of crocin were shown to be associated with its ability to regulate the p38 MAPK/NF-κB/IκBα and Bcl-2/Bax signalling pathways and the reduction of levels of pro-inflammatory cytokines TNF-α and IL-6. For therapeutic strategies, these results suggest that crocin is a potential agent for the treatment of CLP-induced sepsis, owing to its anti-inflammatory and anti-apoptotic activities. The results also provide support for pursuing therapeutic approaches that target the mechanism of action underlying sepsis injury.

## References

[CIT0001] Alavizadeh SH, Hosseinzadeh H. 2014. Bioactivity assessment and toxicity of crocin: a comprehensive review. Food Chem Toxicol. 64:65–80. 24275090 10.1016/j.fct.2013.11.016

[CIT0002] Aziz M, Ode Y, Zhou M, Ochani M, Holodick NE, Rothstein TL, Wang P. 2018. B-1a cells protect mice from sepsis-induced acute lung injury. Mol Med. 24(1):26. 30134811 10.1186/s10020-018-0029-2 PMC6016888

[CIT0003] Baradaran Rahim V, Khammar MT, Rakhshandeh H, Samzadeh-Kermani A, Hosseini A, Askari VR. 2019. Crocin protects cardiomyocytes against LPS-induced inflammation. Pharmacol Rep. 71(6):1228–1234. 31670059 10.1016/j.pharep.2019.07.007

[CIT0004] Boussabbeh M, Ben Salem I, Belguesmi F, Neffati F, Najjar MF, Abid-Essefi S, Bacha H. 2016. Crocin protects the liver and kidney from patulin-induced apoptosis *in vivo*. Environ Sci Pollut Res Int. 23(10):9799–9808. 26856859 10.1007/s11356-016-6195-2

[CIT0005] Brahmbhatt S, Gupta A, Sharma AC. 2005. Bigendothelin-1 (1-21) fragment during early sepsis modulates tau, p38-MAPK phosphorylation and nitric oxide synthase activation. Mol Cell Biochem. 271(1–2):225–237. 15881674 10.1007/s11010-005-6416-3

[CIT0006] Cao WQ, Zhai XQ, Ma JW, Fu XQ, Zhao BS, Zhang P, Fu XY. 2020. Natural borneol sensitizes human glioma cells to cisplatin-induced apoptosis by triggering ROS-mediated oxidative damage and regulation of MAPKs and PI3K/AKT pathway. Pharm Biol. 58(1):72–79. 31875760 10.1080/13880209.2019.1703756 PMC6970185

[CIT0007] Chang JC. 2019. Sepsis and septic shock: endothelial molecular pathogenesis associated with vascular microthrombotic disease. Thromb J. 17:10. 31160889 10.1186/s12959-019-0198-4 PMC6542012

[CIT0008] Chen HG, Han HZ, Li Y, Yu YH, Xie KL. 2020. Hydrogen alleviated organ injury and dysfunction in sepsis: the role of cross-talk between autophagy and endoplasmic reticulum stress: experimental research. Int Immunopharmacol. 78:106049. 31830624 10.1016/j.intimp.2019.106049

[CIT0009] Chhimwal J, Sharma S, Kulurkar P, Patial V. 2020. Crocin attenuates CCl_4_-induced liver fibrosis via PPAR-γ mediated modulation of inflammation and fibrogenesis in rats. Hum Exp Toxicol. 39(12):1639–1649. 32633567 10.1177/0960327120937048

[CIT0010] Cho W, Koo JY, Park Y, Oh K, Lee S, Song JS, Bae MA, Lim D, Lee DS, Park SB. 2017. Treatment of sepsis pathogenesis with high mobility group box protein 1-regulating anti-inflammatory agents. J Med Chem. 60(1):170–179. 28001381 10.1021/acs.jmedchem.6b00954

[CIT0011] Chousterman BG, Swirski FK, Weber GF. 2017. Cytokine storm and sepsis disease pathogenesis. Semin Immunopathol. 39(5):517–528. 28555385 10.1007/s00281-017-0639-8

[CIT0012] El-Kharrag R, Amin A, Hisaindee S, Greish Y, Karam SM. 2017. Development of a therapeutic model of precancerous liver using crocin-coated magnetite nanoparticles. Int J Oncol. 50(1):212–222. 27878253 10.3892/ijo.2016.3769

[CIT0013] Englert JA, Bobba C, Baron RM. 2019. Integrating molecular pathogenesis and clinical translation in sepsis-induced acute respiratory distress syndrome. JCI Insight. 4(2):e124061. 10.1172/jci.insight.124061 PMC6413834 30674720

[CIT0014] Fu H, Wang QS, Luo Q, Tan S, Su H, Tang SL, Zhao ZL, Huang LP. 2014. Simvastatin inhibits apoptosis of endothelial cells induced by sepsis through upregulating the expression of Bcl-2 and downregulating Bax. World J Emerg Med. 5(4):291–297. 25548604 10.5847/wjem.j.issn.1920-8642.2014.04.009 PMC4272934

[CIT0015] Gharamti A, Samara O, Monzon A, Scherger S, DeSanto K, Sillau S, Franco-Paredes C, Henao-Martinez A, Shapiro L. 2021. Association between cytokine levels, sepsis severity and clinical outcomes in sepsis: a quantitative systematic review protocol. BMJ Open. 11(8):e048476. 10.1136/bmjopen-2020-048476 PMC8354287 34373304

[CIT0016] Gille-Johnson P, Hansson KE, Gardlund B. 2013. Severe sepsis and systemic inflammatory response syndrome in emergency department patients with suspected severe infection. Scand J Infect Dis. 45(3):186–193. 23113708 10.3109/00365548.2012.720025

[CIT0017] Guo W, Liu W, Chen G, Hong S, Qian C, Xie N, Yang X, Sun Y, Xu Q. 2012. Water-soluble andrographolide sulfonate exerts anti-sepsis action in mice through down-regulating p38 MAPK, STAT3 and NF-κB pathways. Int Immunopharmacol. 14(4):613–619. 23036579 10.1016/j.intimp.2012.09.002

[CIT0018] Hadipour M, Meftahi GH, Afarinesh MR, Jahromi GP, Hatef B. 2021. Crocin attenuates the granular cells damages on the dentate gyrus and pyramidal neurons in the CA3 regions of the hippocampus and frontal cortex in the rat model of Alzheimer's disease. J Chem Neuroanat. 113:101837. 32534024 10.1016/j.jchemneu.2020.101837

[CIT0019] Heung M, Koyner JL. 2015. Entanglement of sepsis, chronic kidney disease, and other comorbidities in patients who develop acute kidney injury. Semin Nephrol. 35(1):23–37. 25795497 10.1016/j.semnephrol.2015.01.004

[CIT0020] Hu W, Gao W, Miao J, Xu Z, Sun L. 2021. Alamandine, a derivative of angiotensin-(1-7), alleviates sepsis-associated renal inflammation and apoptosis by inhibiting the PI3K/Ak and MAPK pathways. Peptides. 146:170627. 34400214 10.1016/j.peptides.2021.170627

[CIT0021] Huang B, He D, Chen G, Ran X, Guo W, Kan X, Wang W, Liu D, Fu S, Liu J. 2018. α-Cyperone inhibits LPS-induced inflammation in BV-2 cells through activation of Akt/Nrf2/HO-1 and suppression of the NF-κB pathway. Food Funct. 9(5):2735–2743. 29667667 10.1039/c8fo00057c

[CIT0022] Huang M, Cai S, Su J. 2019. The pathogenesis of sepsis and potential therapeutic targets. Int J Mol Sci. 20(21):5376. 10.3390/ijms20215376 PMC6862039 31671729

[CIT0023] Hung YL, Fang SH, Wang SC, Cheng WC, Liu PL, Su CC, Chen CS, Huang MY, Hua KF, Shen KH, et al. 2017. Corylin protects LPS-induced sepsis and attenuates LPS-induced inflammatory response. Sci Rep. 7:46299. 28397806 10.1038/srep46299 PMC5387730

[CIT0024] Kalantar M, Kalantari H, Goudarzi M, Khorsandi L, Bakhit S, Kalantar H. 2019. Crocin ameliorates methotrexate-induced liver injury via inhibition of oxidative stress and inflammation in rats. Pharmacol Rep. 71(4):746–752. 31220735 10.1016/j.pharep.2019.04.004

[CIT0025] Khanmohammadi F, Shahrooz R, Ahmadi A, Razi M. 2021. Possible protective effects of crocin on destructive side effects of cyclo-phosphamide in mice ovarian tissue: evaluation of histomorphometrical and biochemical changes. Vet Res Forum. 12(2):217–222. 34345389 10.30466/vrf.2019.103192.2453 PMC8328250

[CIT0026] Kim JH, Park GY, Bang SY, Park SY, Bae SK, Kim Y. 2014. Crocin suppresses LPS-stimulated expression of inducible nitric oxide synthase by upregulation of heme oxygenase-1 via calcium/calmodulin-dependent protein kinase 4. Mediators Inflamm. 2014:728709. 24839356 10.1155/2014/728709 PMC4009253

[CIT0027] Klingensmith NJ, Fay KT, Lyons JD, Chen CW, Otani S, Liang Z, Chihade DB, Burd EM, Ford ML, Coopersmith CM. 2019. Chronic alcohol ingestion worsens survival and alters gut epithelial apoptosis and CD8^+^ T cell function after *Pseudomonas aeruginosa* pneumonia-induced sepsis. Shock. 51(4):453–463. 29664837 10.1097/SHK.0000000000001163 PMC6191382

[CIT0028] Kocaman G, Altinoz E, Erdemli ME, Gul M, Erdemli Z, Gul S, Bag HG. 2019. Protective effects of crocin on biochemistry and histopathology of experimental periodontitis in rats. Biotech Histochem. 94(5):366–373. 30982354 10.1080/10520295.2019.1571229

[CIT0029] Lari P, Abnous K, Imenshahidi M, Rashedinia M, Razavi M, Hosseinzadeh H. 2015. Evaluation of diazinon-induced hepatotoxicity and protective effects of crocin. Toxicol Ind Health. 31(4):367–376. 23406950 10.1177/0748233713475519

[CIT0030] Li R, Ren T, Zeng J. 2019. Mitochondrial coenzyme Q protects sepsis-induced acute lung injury by activating PI3K/Akt/GSK-3β/mTOR pathway in rats. Biomed Res Int. 2019:5240898. 31815144 10.1155/2019/5240898 PMC6878790

[CIT0031] Lin GL, McGinley JP, Drysdale SB, Pollard AJ. 2018. Epidemiology and immune pathogenesis of viral sepsis. Front Immunol. 9:2147. 30319615 10.3389/fimmu.2018.02147 PMC6170629

[CIT0032] Lin L, Liu G, Yang L. 2019. Crocin improves cognitive behavior in rats with Alzheimer's disease by regulating endoplasmic reticulum stress and apoptosis. Biomed Res Int. 2019:1–9. 10.1155/2019/9454913 PMC6732583 31534969

[CIT0033] Malkoç M, Patan H, Yaman SÖ, Türedi S, Kerimoğlu G, Kural BV, Örem A. 2020. l-Theanine alleviates liver and kidney dysfunction in septic rats induced by cecal ligation and puncture. Life Sci. 249:117502. 32142764 10.1016/j.lfs.2020.117502

[CIT0034] Mashmoul M, Azlan A, Mohtarrudin N, Mohd Yusof BN, Khaza'ai H, Khoo HE, Farzadnia M, Boroushaki MT. 2016. Protective effects of saffron extract and crocin supplementation on fatty liver tissue of high-fat diet-induced obese rats. BMC Complement Altern Med. 16(1):401. 27770798 10.1186/s12906-016-1381-9 PMC5075149

[CIT0035] Minasyan H. 2017. Sepsis and septic shock: pathogenesis and treatment perspectives. J Crit Care. 40:229–242. 28448952 10.1016/j.jcrc.2017.04.015

[CIT0036] Naghizadeh B, Boroushaki MT, Vahdati Mashhadian N, Mansouri MT. 2008. Protective effects of crocin against cisplatin-induced acute renal failure and oxidative stress in rats. Iran Biomed J. 12(2):93–100. 18506215

[CIT0037] Omidkhoda SF, Mehri S, Heidari S, Hosseinzadeh H. 2020. Protective effects of crocin against hepatic damages in d-galactose aging model in rats. Iran J Pharm Res. 19:440–450. 33680043 10.22037/ijpr.2019.15022.12825 PMC7757971

[CIT0038] Razmaraii N, Babaei H, Mohajjel Nayebi A, Assadnassab G, Ashrafi Helan J, Azarmi Y. 2016. Crocin treatment prevents doxorubicin-induced cardiotoxicity in rats. Life Sci. 157:145–151. 27297631 10.1016/j.lfs.2016.06.012

[CIT0039] Rello J, Valenzuela-Sanchez F, Ruiz-Rodriguez M, Moyano S. 2017. Sepsis: a review of advances in management. Adv Ther. 34(11):2393–2411. 29022217 10.1007/s12325-017-0622-8 PMC5702377

[CIT0040] Teoh H, Quan A, Creighton AK, Annie Bang KW, Singh KK, Shukla PC, Gupta N, Pan Y, Lovren F, Leong-Poi H, et al. 2013. BRCA1 gene therapy reduces systemic inflammatory response and multiple organ failure and improves survival in experimental sepsis. Gene Ther. 20(1):51–61. 22257935 10.1038/gt.2011.214

[CIT0041] Vafaei S, Motejaded F, Ebrahimzadeh-Bideskan A. 2020. Protective effect of crocin on electromagnetic field-induced testicular damage and heat shock protein A2 expression in male BALB/c mice. Iran J Basic Med Sci. 23(1):102–110. 32395207 10.22038/IJBMS.2019.38896.9229 PMC7206838

[CIT0042] Wang Z, Chen Z, Li B, Zhang B, Du Y, Liu Y, He Y, Chen X. 2020. Curcumin attenuates renal interstitial fibrosis of obstructive nephropathy by suppressing epithelial-mesenchymal transition through inhibition of the TLR4/NF-кB and PI3K/AKT signalling pathways. Pharm Biol. 58(1):828–837. 10.1080/13880209.2020.1809462 PMC7470153 32866059

[CIT0043] Yaribeygi H, Mohammadi MT, Sahebkar A. 2018. Crocin potentiates antioxidant defense system and improves oxidative damage in liver tissue in diabetic rats. Biomed Pharmacother. 98:333–337. 29274590 10.1016/j.biopha.2017.12.077

[CIT0044] Yu YQ, He XR, Wan LJ, Yang YH, Chen PY. 2021. Etiology, antimicrobial resistance, and risk factors of neonatal sepsis in China: a systematic review and meta-analysis from data of 30 years. J Matern Fetal Neonatal Med. 1–10. 10.1080/14767058.2021.1951217 34470123

[CIT0045] Zaghloul MS, Said E, Suddek GM, Salem HA. 2019. Crocin attenuates lung inflammation and pulmonary vascular dysfunction in a rat model of bleomycin-induced pulmonary fibrosis. Life Sci. 235:116794. 31465731 10.1016/j.lfs.2019.116794

